# Molecular Mechanics of the α-Actinin Rod Domain: Bending, Torsional, and Extensional Behavior

**DOI:** 10.1371/journal.pcbi.1000389

**Published:** 2009-05-15

**Authors:** Javad Golji, Robert Collins, Mohammad R. K. Mofrad

**Affiliations:** Molecular Cell Biomechanics Laboratory, University of California Berkeley, Berkeley, California, United States of America; University of Houston, United States of America

## Abstract

α-Actinin is an actin crosslinking molecule that can serve as a scaffold and maintain dynamic actin filament networks. As a crosslinker in the stressed cytoskeleton, α-actinin can retain conformation, function, and strength. α-Actinin has an actin binding domain and a calmodulin homology domain separated by a long rod domain. Using molecular dynamics and normal mode analysis, we suggest that the α-actinin rod domain has flexible terminal regions which can twist and extend under mechanical stress, yet has a highly rigid interior region stabilized by aromatic packing within each spectrin repeat, by electrostatic interactions between the spectrin repeats, and by strong salt bridges between its two anti-parallel monomers. By exploring the natural vibrations of the α-actinin rod domain and by conducting bending molecular dynamics simulations we also predict that bending of the rod domain is possible with minimal force. We introduce computational methods for analyzing the torsional strain of molecules using rotating constraints. Molecular dynamics extension of the α-actinin rod is also performed, demonstrating transduction of the unfolding forces across salt bridges to the associated monomer of the α-actinin rod domain.

## Author Summary

The cell interacts with its environment in both biochemical and mechanical ways. In this study we explore one of the ways in which the cell interacts mechanically with its environment. α-Actinin is a cytoskeletal crosslinker: it functions to scaffold the cytoskeletal actin filaments that provide mechanical reinforcement to the cell. In its functional environment α-actinin is exposed to a multitude of mechanical stresses as it attaches itself to a dynamic network of actin filaments. The actin filaments extend, rotate, and bend the α-actinin crosslinkers. In this study we employ molecular dynamics techniques to understand the structural characteristics of α-actinin that underlie its ability to provide a scaffold in such a stressed environment. We analyzed the natural frequencies of α-actinin and simulated force-induced bending, extension, and twisting. Our results suggest that α-actinin has structural flexibility facilitating crosslinking in a dynamic environment and also structural rigidity stabilizing the linkage in the stressed environment. We have discovered novel natural bending movements of the rod domain that enhance its function as a crosslinker. We have also demonstrated the specific structural characteristics of α-actinin that give it the previously suggested property of having partial flexibility. Our results enhance the understanding of structural mechanics in the cytoskeletal molecules.

## Introduction

Cytoskeletal microfilament networks contribute to the mechanical stability of the cell by dynamically arranging and rearranging actin filaments for reinforcement. The dynamic arrangement of actin filament requires actin filament crosslinking molecules such as α-actinin. α-Actinin is a 200 kDa homodimer with three major structural motifs: the actin binding domain (ABD), the calmodulin homology domain (Cam), and the central rod domain [1]. Each monomer contains all three structural domains but the two monomers are arranged anti-parallel so that the two ABDs are at opposite ends of α-actinin. The arrangement of the two ABDs at opposite ends allows for α-actinin to crosslink parallel actin filaments [2]. Actin filaments in the parallel arrangement are very dynamic; the actin filaments move laterally and horizontally in relationship to each other, and continuously bind and unbind α-actinin crosslinking molecules [3]. Several cellular processes involving actin filament dynamic rearrangement and scaffolding by α-actinin include: focal adhesion formation near membrane bound integrin molecules [4], cytokinesis and cytoplasmic dumping in the final stages of mitosis [5],[6], and z-disk formation and stabilization in muscle cells [7]. In order for α-actinin to maintain its function as an actin filament scaffold in such a dynamic environment, the α-actinin molecule must be partially flexible, meaning it must simultaneously be rigid and stable at some regions to resist external stress and be flexible at other regions to maintain binding in a dynamic environment [8]–[10].

**Figure 1:**
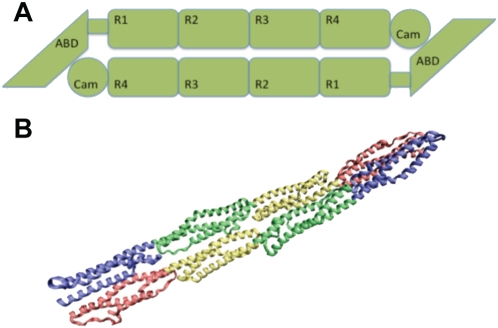
The α-actinin structure. A) α-actinin is a dimer with three major domains: an actin binding domain, a calmodulin homology domain, and a central rod domain. The monomers are arranged in an anti-parallel manner. B) VMD [52] generated image of the α-actinin dimer rod domain. Each of the four spectrin repeats are colored according to conformation. R1 is colored in red, R2 in yellow, R3 in green, and R4 in blue. In dimer conformation R1 is interacting with R4 and R2 is interacting with R3.

Structure of the α-actinin rod domain underlies the function of α-actinin as a partially flexible actin filament crosslinker. Each central rod domain monomer is 240 Å long and made up of 4 spectrin (R1–R4) repeats connected by helical linkers (see Figure 1) [11],[12]. Other molecules with spectrin repeats include dystophin and utrophin. The α-actinin rod domain differs from the other spectrin family molecules by its shorter length, its more rigid helical linkers, and its dimerization [13]. The spectrin repeats structure of the rod domain contributes several vital characteristics to the α-actinin rod domain: aromatic packing and hydrophobic residues within each repeat stabilize secondary structure [8]; acidic and basic surfaces on R1 and R4 confer strong dimerization interactions [1], Kd of 10 pM between monomers [14]; interaction of hydrophobic residues between R2 and R3 on both monomers and electrostatic interactions produce a coiled-coil homodimer conformation with a 12 degree bend and a 90 degree left handed twist [15]. Together these characteristics account for the rod domain maintaining both structural rigidity and flexibility.

The goal of this investigation is to understand the structural mechanisms of the partial flexibility of the α-actinin rod domain. The coiled-coil nature of the rod domain is an essential component of the rod domain structure. Coiled-coils are the dominant conformation for fibrous proteins [16]. Most coiled-coils have a heptad conformation, with hydrophobic residues every seventh residue [17],[18]. The heptad conformation allows for hydrophobic insertion of one linker region into that of the other monomers by a knobs-into-holes mechanism [19]. The presence of heptad hydrophobic residues is common in coiled-coil structure but neither necessary nor sufficient [17],[18]. Coiled-coils with antiparallel dimers like the α-actinin rod domain are stabilized mainly by electrostatic interactions between the monomers, and within the monomers [20]. In general the knobs-into-holes mechanism of coiled-coil conformation exists only when stabilized by electrostatic interactions [21]. The tendency of electrostatic interactions to play a key role in stabilizing coiled-coil dimers like α-actinin is in contrast to globular proteins, where hydrophobic, VDW, and electrostatic interactions are equally significant to molecular stability [22]. The coiled-coil conformation of the rod domain is a significant structural feature, and the significance of electrostatic interactions to coiled-coil structure stability suggests a significant role of electrostatic interactions in mechanical properties of the α-actinin rod domain.

Several studies have examined the mechanical properties of other molecules with rod-like coiled-coil conformations. These studies on DNA [23]–[26], myosin [27]–[29], and keratin [30],[31] together suggest the coiled-coil rod like structure contributes extensible rigidity and torsional and bending flexibility. The tertiary structure of DNA is referred to as coiled-coil, and more commonly as a double-helix, because it consists of two intertwined α-helices. In contrast, α-actinin and other fibrous proteins are referred to as coiled-coil due to intertwining in their quaternary structures. The difference between the DNA coiled-coil conformation and the protein coiled-coil conformation is significant, but the mechanical properties can still be compared. DNA is the most studied of the coiled-coil conformations and has been described as an elastic rod [32]. Its global mechanical behavior has been described as like a thin isotropic homogeneous rod, but its local mechanical behavior has been described as like an anisotropic heterogeneous rod with bending and torsional flexibility [23]–[26]. Myosin has an S2 region that functions as a lever arm in muscle sarcomeres. Using a single molecule assay in a total internal reflection microscopy experiment [27], it has been shown that the S2 region has significant torsional flexibility underlying its lever arm function. Keratin, the first coiled-coil structure to be discovered [33], is the major molecule in hair fibers, and investigation of its mechanical behavior with molecular dynamics has shown that it has strong stretching rigidity, over 1 nN of force is needed to stretch keratin 90% [30]. Removing the electrostatic interactions underlying the coiled-coil conformation of keratin significantly reduces its rigidity [31]. These studies suggest that the coiled-coil conformation in α-actinin contributes extension rigidity but torsional and bending flexibility.

Studies of α-actinin and other spectrin repeat molecules have similarly demonstrated extension rigidity of the coiled-coil rod domain [34]–[37]. Experimental investigation using atomic force microscopy (AFM) of spectrin unfolding demonstrated that spectrin repeats unfold in a cooperative mechanism [35]. Several molecular dynamics investigations further characterize the extension rigidity of the α-actinin rod domain as resulting from the strength of the helical linker between the spectrin repeats, and electrostatic and hydrogen bonding within each repeat [31]–[34]. There has been no investigation of the bending or torsional flexibility of the α-actinin rod domain or other spectrin repeats, but investigation of α-actinin structure using cryoelectron microscopy has shown that there must be some structural flexibility since α-actinin molecules form stable actin filaments crosslinks in a range of crosslinking angles [38]. Is the flexibility of α-actinin in crosslinking actin filaments due to torsional and bending flexibility of the rod domain? What features of the coiled-coil structure of α-actinin underlie its partial flexibility?

Using molecular dynamics and normal mode analysis this study investigates the mechanical partial flexibility of the α-actinin rod domain. Bending, torsion and extension simulations demonstrate that, as with other coiled-coil molecules, the α-actinin rod domain has bending and torsional flexibility and extensional rigidity. Normal mode analysis shows that the rod-like structure of α-actinin contributes towards its bending and torsional flexibility. Our simulations suggest that aromatic packing interactions determine the trajectory of torsion on the rod domain, and that electrostatic interaction between the monomers contributes extension rigidity to the rod domain.

## Results

### Normal Mode Analysis

Structural properties of α-actinin can be inferred from its natural vibrations, therefore, to reveal the naturally rigid and flexible regions of the α-actinin rod domain, we carried out normal mode analysis (NMA). Results from NMA convey properties inherent in the structure of α-actinin regardless of what intermolecular interactions are present [39]. The purpose of our NMA is to determine the contributions of the α-actinin rod-like structure to its mechanical behavior. Later sections will investigate contributions of intermolecular interactions to its mechanical behavior. NMA was carried out on both the monomer conformation of the rod domain containing only four spectrin repeats and on the dimer conformation containing eight total spectrin repeats arranged in an anti-parallel coiled-coil conformation. The NMA results suggest the α-actinin rod domain to have natural bending and torsional flexibility.

**Figure 2:**
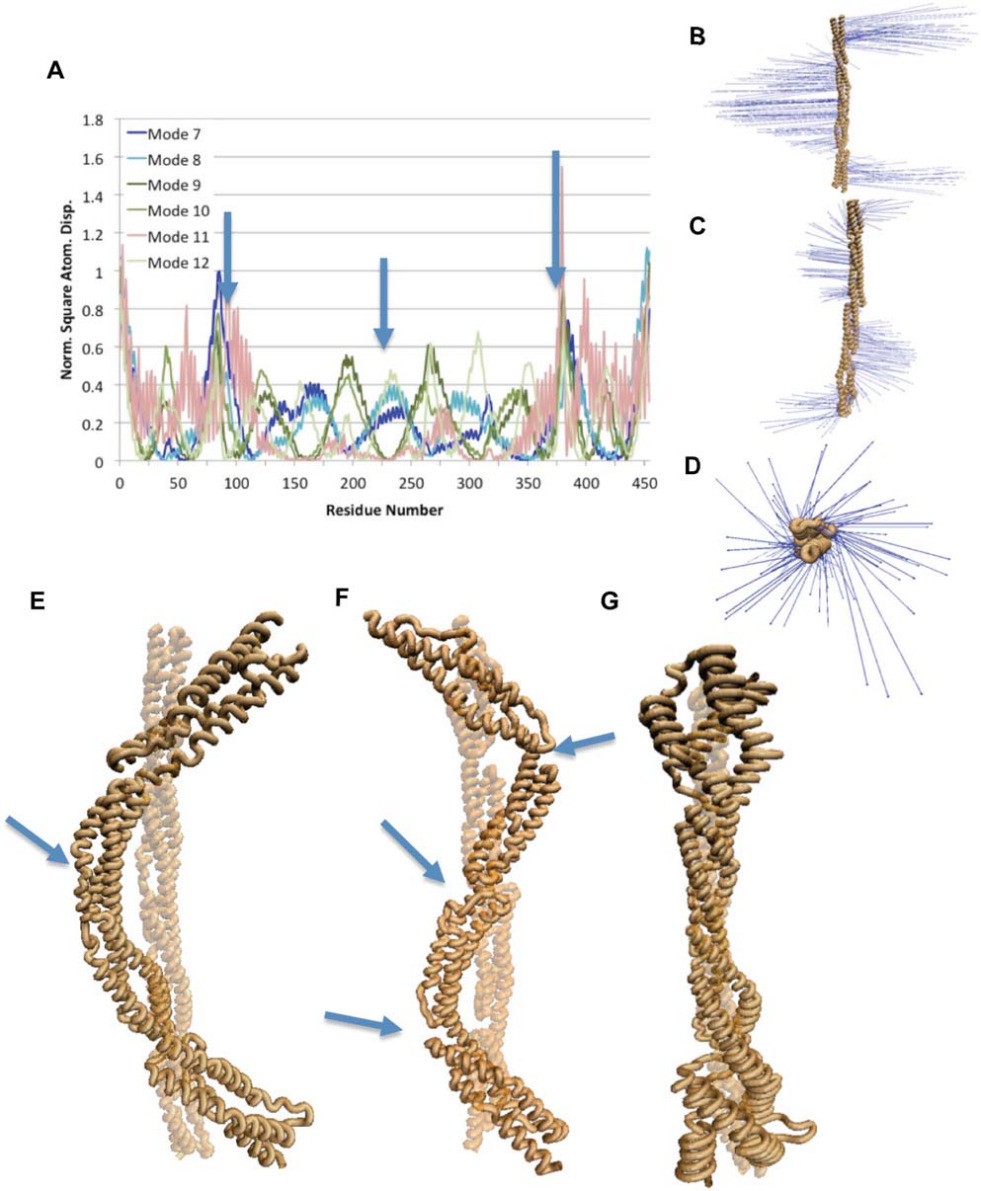
Normal mode analysis of the α-actinin monomer revealed bending and torsional natural frequencies. A) RMSD of individual residues for six lowest vibrational modes. Natural movements consisted mainly of movement in the regions near the termini. Single-hinge bending modes are shown in blue colors, three-hinge bending modes in green colors, and the torsional mode is shown in pink. Arrows indicate location of hinges. B) Vector field representation of movements during one-hinge bending normal modes. Vector field representations show the direction and magnitude of movement of each residue in the molecule. Larger vectors represent larger movements. Mode 7 and mode 8 exhibited this type of bending motion. C) Vector field representation of movements during three-hinge bending modes. Mode 9, mode 10, and mode 12 showed three-hinge bending movement. D) Vector field representation of movement in the torsional mode. In the α-actinin monomer normal mode analysis only mode 11 showed torsional movement. E) Image captures movement characteristic of the single-hinge bending modes (7 and 8). Arrow points to location of the single-hinge. F) Image captures movement characteristic of the three-hinge bending modes (9, 10, and 12). Arrows point to location of the three hinges. G) Image showing the torsional movement in mode 11. Images were rendered using VMD [52]. RMSD plots were created using WEBnm@ [47].

NMA from a single monomer of α-actinin suggested that the monomer has significant bending flexibility and some torsional flexibility. The six lowest frequencies of α-actinin are rotational and translational modes. Mode 7 and mode 8, the two lowest frequency vibrational modes, show bending movement mainly at the termini with a single hinge at the central linker (Figure 2 A,B, and E). Of the other lowest frequency vibrational modes, three modes: 9, 10, and 12, exhibit bending modes with three hinge regions, each at the three linker regions between the spectrin repeats (Figure 2 A, C, and F). A three-hinge bending movement refers to bending with three different hinges, resulting in the molecule being divided into four sections, each undergoing movement in different directions. The other lowest frequency normal mode, mode 11, exhibits torsional movement (Figure 2 A, D, G, and Video S1). The torsional motion in mode 11 is localized to the regions near the termini. The α-actinin monomer NMA results suggest bending and torsion movements to be natural movements, movements that are exhibited by the natural vibrational frequencies, and reveal the residues near the termini to be more flexible and the linker residues to be more rigid.

**Figure 3:**
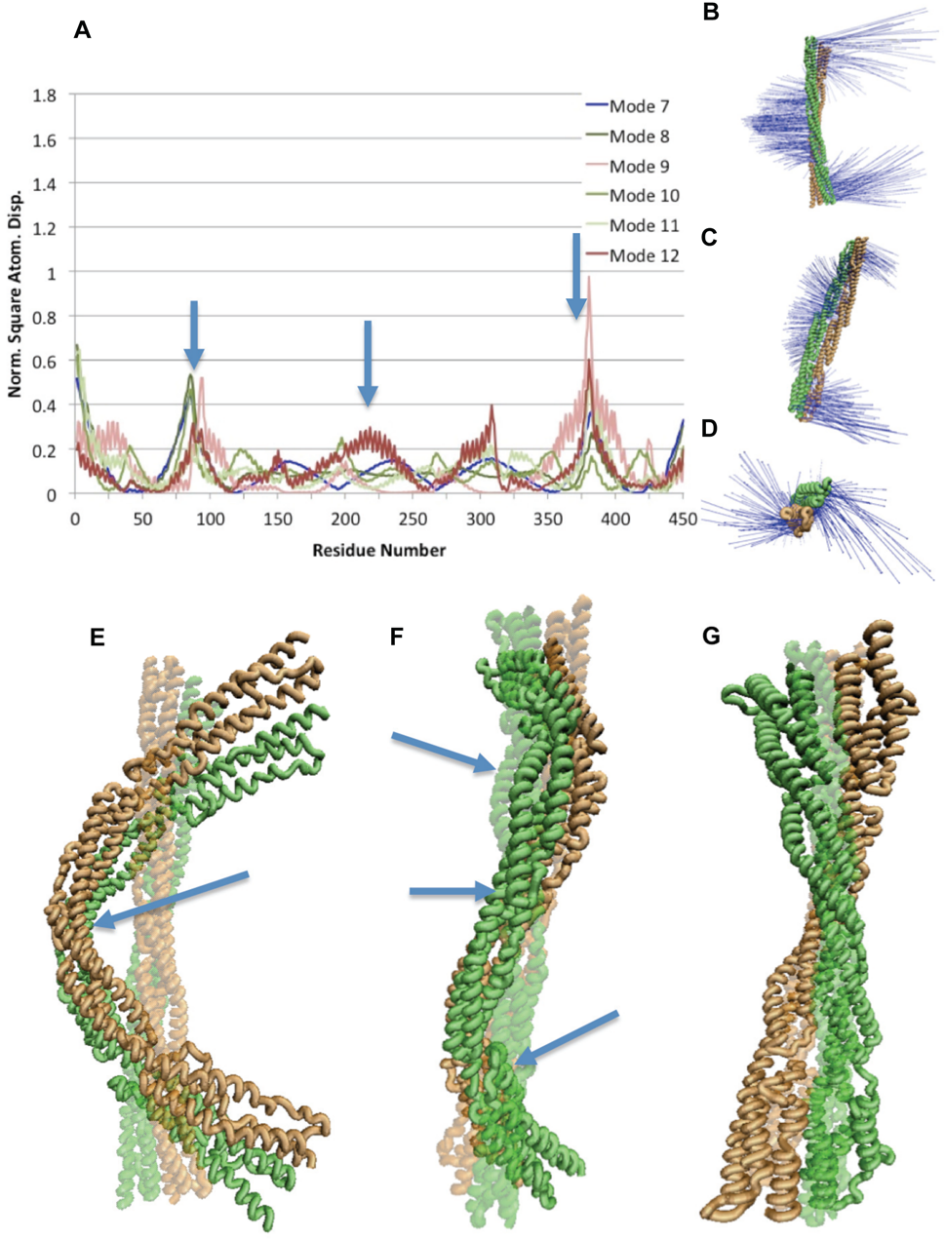
Natural frequencies of the α-actinin dimer. Normal mode analysis using WEBnm@ [47] on the α-actinin dimer revealed modes with bending, twisting, and both motions. A) Six lowest vibrational modes of the α-actinin dimer shown with RMSD analysis of individual residues. In all modes vibrations are seen mainly at the termini (near residues 1, residues 475, and residues 950). Plot of single-hinge bending modes are shown in blue colors, three-hinge bending and torsion modes shown in green colors, and torsional modes shown in red colors. Arrows indicate location of hinges. B) Vector field representation of bending vibrational motion in mode 7. C) Vector field representation of the simultaneous bending and twisting vibrations in modes 8, 10, and 11. D) Vector field representation of torsional vibrations seen in mode 9 and 12. E) VMD [52] rendered image of the vibration in mode 7 characteristic of bending. Arrow points to single-hinge in bending mode. F) Bending and twisting motion, at the same time, shown here in a VMD representation. Modes 8,10, and 11 exhibit simultaneous bending and twisting. Arrows point to location of three hinges. G) The torsional motion of modes 9 and 12 captured in VMD. Most of the torsional natural vibration is at the termini.

NMA of the rod domain dimer suggested that the bending flexibility is retained in dimerization, while a new natural movement involving torsion and bending is present (Figure 3 C and F). The lowest vibrational frequency for the dimerized α-actinin rod domain (mode 7) is a single-hinge bending mode with hinge action at the central repeat as with the monomer (Figure 3 A, B, and E). The other bending modes, modes 8, 10, and 11, in the dimer also show torsional motion along with the bending motion (Figure 3 A, C, and F). Mode 8 showed the most pronounced bending and torsion, while modes 10 and 11 showed similar characteristics with more subtle movements. Two torsional modes exist for the dimer conformation, modes 9 and 12 (Figure 3 A, D, G, and Video S2). The torsional movement is localized to the termini as with the NMA of the α-actinin monomer. Dimerization maintained natural vibrations of bending and torsion, and still exhibited flexibility near the termini of the α-actinin rod domain. Vibrational movement in several additional normal modes for both the monomer and dimer are listed in Table S1.

### Molecular Dynamics Simulation of Bending

**Figure 4:**
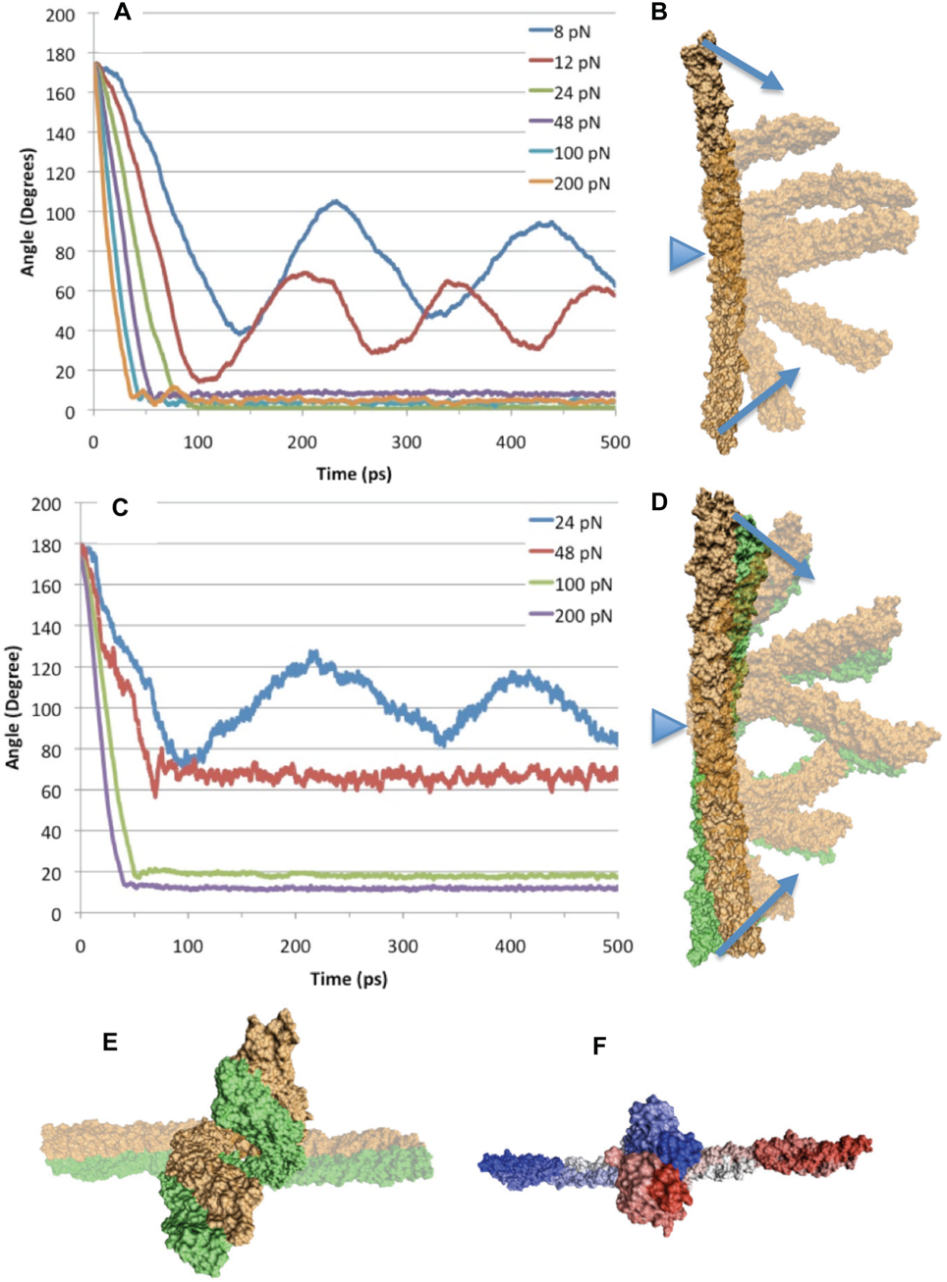
Force induced bending of α-actinin. Forced bending molecular dynamics simulations were carried out using CHARMm [54]. A) Total bending forces ranging from 8 to 200 pN were applied to the α-actinin rod domain monomer. Forces less than 24 pN (8 pN shown in blue and 12 pN shown in red) failed to fully bend the monomer. The rate of bending is directly proportional to the total bending force. Trajectory for total force values of 24 pN shown in green, 48 pN shown in purple, 100 pN shown in blue, and 200 pN shown in orange. Graph shows angle between two termini and the central hinge throughout the simulation. B) Bending path of the α-actinin monomer. Successive steps were superimposed on the original conformation and represented using transparent material in VMD [52]. Arrows indicate the direction of bending force applied to the ends of the rod. Triangle indicates location of hinge. C) Graph showing trajectory of bending simulations on the dimer conformation of the α-actinin rod domain. Forces show as: 24 pN is blue, 48 pN is red, 100 pN is green, and 200 pN is purple. Only forces of 100 pN and 200 pN achieved total bending. D) Trajectory of bending of the dimer conformation of the α-actinin rod domain. Successive steps from the bending simulation have been superimposed using VMD. Arrows indicate the direction of bending force applied to the ends of the rod. Triangle indicates location of hinge. E) Side view of fully bent dimer conformation superimposed on a transparent unbent α-actinin dimer. The bending results in two termini being side by side not on top of each other because of the 90-degree coiled-coil conformation. F) Index coloring representation of the α-actinin rod domain monomer. Terminus B (red) rotates underneath terminus A (blue) after bending is completed in a natural coiled-coil conformation.

The above results (Figures 2 and 3) indicate bending to be the most natural movement for the α-actinin rod domain since the lowest natural frequency vibration of the α-actinin rod in both monomer and dimer conformations was a single-hinge bending motion. To test normal mode analysis findings and to determine the mechanical consequence of having bending be the natural normal mode for α-actinin, we simulated bending using constant force molecular dynamics simulations (Figure 4). The simulations suggested that while the α-actinin rod domain has bending flexibility, dimerization of the α-actinin rod domain enhances the bending rigidity.

Simulation of bending of the monomer suggested bending flexibility. Interestingly, the bent monomer arranged itself into a coiled-coil structure. Total force as low as 24 pN was able to achieve complete bending of the α-actinin rod domain monomer (Figure 4A). The bending simulation showed initial movement by repeats (R1 and R4) followed by movement by the other two repeats and swift collapse of the two ends together (Figure 4B). Once bending of the α-actinin rod domain monomer to zero degrees between the termini was completed, the molecule proceeded to adopt a coiled-coil conformation similar to the dimer conformation, only two spectrin repeats in length not four (Figure 4F). The C-terminus spectrin repeat moves from being in plane with the central linker and the N-terminus to being in plane with the N-terminus but rotated 90 degrees relative to the central linker. The final conformation of the bent monomer is similar to the conformation of half of the full dimer with R1 and R4 in surface contact, and R2 and R3 in surface contact.

The molecular dynamics simulations under bending forces with the α-actinin dimer showed more resistance to bending than the α-actinin rod domain monomer (Figure 4C). The minimum force required to completely bend the dimer was 100 pN compared to the 24 pN to bend the monomer (Figure 4 A and C). Different force levels also showed different rates of bending (as with the simulations of monomer bending). The trajectory of bending shows non-localized movement of the entire molecule during bending (Figure 4D). Repeats 1 and 4 moved before repeats 2 and 3, but the bending force was later transduced and the entire dimer showed movement together. A side view of the bent dimer (Figure 4E) shows that the two bent halves of the molecule fail to collapse on top of each other because of the 90 degree coiled-coil conformation in the dimer conformation; the molecule halves sit next to each other. Further supercoiling is not seen upon bending as in the monomer simulations since R1 and R4 are already in surface contact at both ends of the dimer, and there are no exposed complementary surfaces or unsatisfied salt bridges to interact and coil.

### Molecular Dynamics Simulation of Torsion

**Figure 5:**
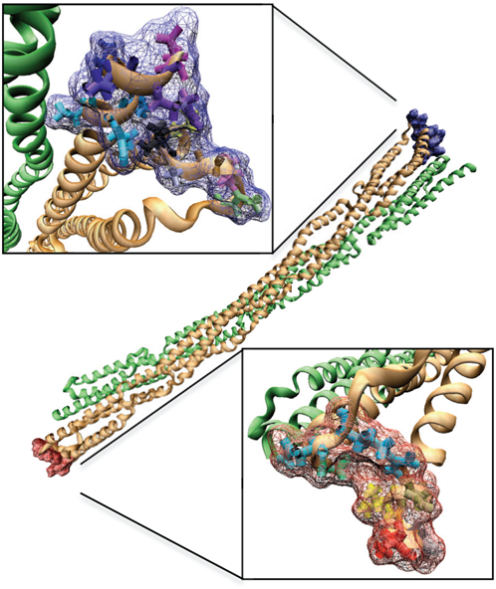
Torque was applied at the termini of the α-actinin rod domain. Α-actinin rod domain dimer shown with twisted monomer colored in orange and the other monomer colored in green. Constraints were placed on terminus B and terminus A. For rotations at terminus B, terminus A residues were constrained fixed while terminus B residues were constrained to rotate, and visa versa for rotation at terminus A. At terminus B (upper panel) constraints are placed on residues: 396 (light green), 397 (light pink), 398 (orange), 399 (gray), 400 (black), 401 (yellow), 469 (light blue-green), 470 (blue-green), 471 (light blue), 472 (dark blue), 473 (dark purple), 474 (light purple), 475 (dark pink). At terminus A (lower panel) constraints are placed on residues: 1 (red), 2 (black), 3 (orange), 4 (yellow), 5 (tan), 84 (light green), 85 (light blue-green), 86 (blue-green).

Using rotating constraints, we implemented a molecular dynamics study of torsion induced conformational changes in the α-actinin rod domain. Torque was applied first to a single monomer of the rod domain at both the N-terminus and C-terminus, and also to a single monomer in dimer conformation. For comparison between torsional simulations of the monomer and dimer we define terminus A as the terminus with residue 1 (N-terminus in the monomer) and terminus B as the terminus with residue 475 (C-terminus in the monomer) (Figure 5). Torque studies of the dimer involved rotation of terminus A and terminus B of one monomer in dimer conformation. Direction of rotation is defined as either clockwise or counterclockwise with respect to the viewing angle looking along the axis of rotation at the site of torsion. Clockwise rotation at terminus B refers to clockwise rotation if viewed along the axis of rotation at terminus B, and would therefore be seen as counterclockwise if viewed along the axis of rotation from terminus A. To avoid ambiguity, all rotation directions referred to are viewed from the terminus at which the rotation is taking place.

**Figure 6:**
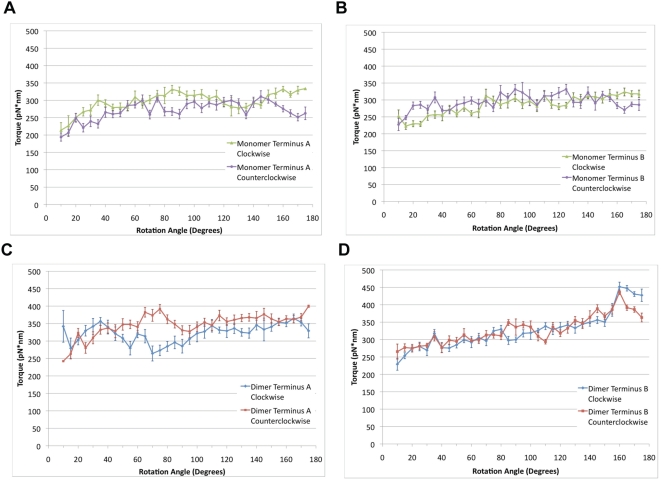
Torque profile of the α-actinin monomer and dimer. Torsion was applied to the termini of both the α-actinin rod domain monomer (A–B) and dimer (C–D) using rotating constraints in NAMD [59]. Torque was applied at both termini in both the clockwise and counterclockwise directions. A) Comparison of torque needed to rotate terminus A in the clockwise (green) and counterclockwise (purple) directions. The data is plotted using window averaging. Average of torque values in each 5-degree window is plotted with the standard error shown in error bars. Rotation beyond 140 degrees in the clockwise direction shows a significant increase in torque required as compared to counterclockwise rotation. B) Window averaged plot of the rotation of terminus B in either the clockwise (green) or counterclockwise (purple) directions. Clockwise rotation beyond 140 degrees required more torque than counterclockwise rotation. C) Window averaged plot of torque required to rotate the α-actinin rod domain dimer in the clockwise (blue) or counterclockwise (red) directions at terminus A. Clockwise rotation requires less torque than counterclockwise rotation because of steric interactions resulting from rotation of R1 in the counterclockwise direction. D) Torque required to rotate the α-actinin rod domain dimer at terminus B in either the clockwise (blue) or counterclockwise (red) directions. Rotation in either direction increases the total torque required, especially passed 150 degrees because of steric interaction with the complementary monomer.

**Figure 7:**
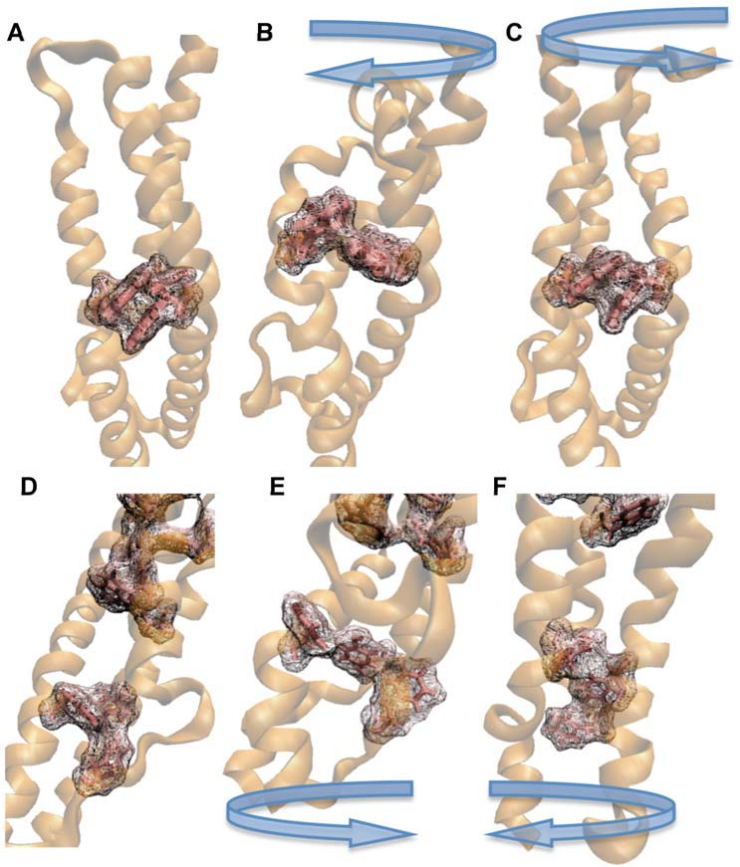
Aromatic packing stabilizes the spectrin repeats under torsion. A) Surface representation of amino acid residues W381, Y417, and W453 near terminus B of a single α-actinin rod domain monomer. Aromatic packing between amino acid residues W381 and Y417 is seen. B) Effect of clockwise rotation of over 140 degrees on the aromatic packing between W381 and Y417. The aromatic packing is disrupted and torque required for continued rotation increases. C) Counterclockwise rotation at terminus B of the monomer conformation does not disrupt aromatic packing, shown here to be intact after rotation. D) More extensive aromatic packing exists at terminus A. Two sets of aromatic residues exist, each set sharing electrons within its members. Both sets are shown here with surface representation in VMD [52]. E) Clockwise rotation of terminus A. Shown here, the aromatic packing in both sets are not completely disrupted, but electron sharing between F89, F74, and Y15 is reduced. F) Counterclockwise rotation of terminus A does not seem to disrupt the aromatic packing shown here as intact with the surface representation. Direction of rotation is indicated above each panel.

The trajectory of the α-actinin monomer under torsional simulation was largely determined by interaction between terminal aromatic residues near the torsional stress. Rotation at both terminus A and terminus B required different levels of torque for the clockwise and the counterclockwise rotation (Figure 6 A and B). Clockwise rotation beyond 140 degrees required significantly more torque than counterclockwise rotation at both terminus A and terminus B. The increasing demand for torque to continue rotation in the clockwise direction is explained by the existence of aromatic packing in the α-actinin rod domain. Aromatic packing describes the arrangement of nearby aromatic residues in either an orientation with the aromatic rings stacked on top of each other or with the edge of one aromatic ring stacked against the face of another [40]. These aromatic packing arrangements are highly stable due to a combination of van der Waals (VDW) interactions, hydrophobic interactions, hydrogen bonding, and electrostatic interactions [41]. Aromatic stacking interactions can be further stabilized by π electron sharing between two stacked aromatic rings [42]. Near terminus B there is interaction between aromatic residues W381, Y417, and W453 (Figure 7A). Clockwise rotation at terminus B disrupted the aromatic packing between these residues after 140 degrees of rotation (Figure 7B and Video S3) and further rotation is thereafter increasingly energetically unfavorable. Rotation of terminus B in the counterclockwise direction failed to disrupt the aromatic packing (Figure 7C) even after 140 degrees of rotation. Similarly, at terminus A there are two sets of aromatic packing interactions. One set immediately near to terminus A is made up of Y15, F74, and F89 (Figure 7D). The other set further back from terminus A but still in repeat R1 is made up of amino acid residues W104, Y25, Y55, W32, F52, Y114, and W117. Although rotation in either clockwise (Figure 7E) or counterclockwise (Figure 7F), direction at terminus A fails to completely disrupt the aromatic packing at these two sites, clockwise rotation does reduce the extent of aromatic packing in the first set nearest the terminus. The decrease in favorable aromatic interactions increases the energetic cost of further rotation and thus requires more torque. Rotation in the counterclockwise direction at both terminus A and terminus B showed a slight decrease in torque required for rotation beyond 140 degrees. The reduction can be explained by free rotation about bonds in adjacent residues and the fact that aromatic packing interactions are not disrupted. Rotation at terminus B shows less sensitivity to the direction of rotation as compared to rotation at terminus A, suggesting a more significant impact of aromatic interactions on stability near terminus A than near terminus B.

**Figure 8:**
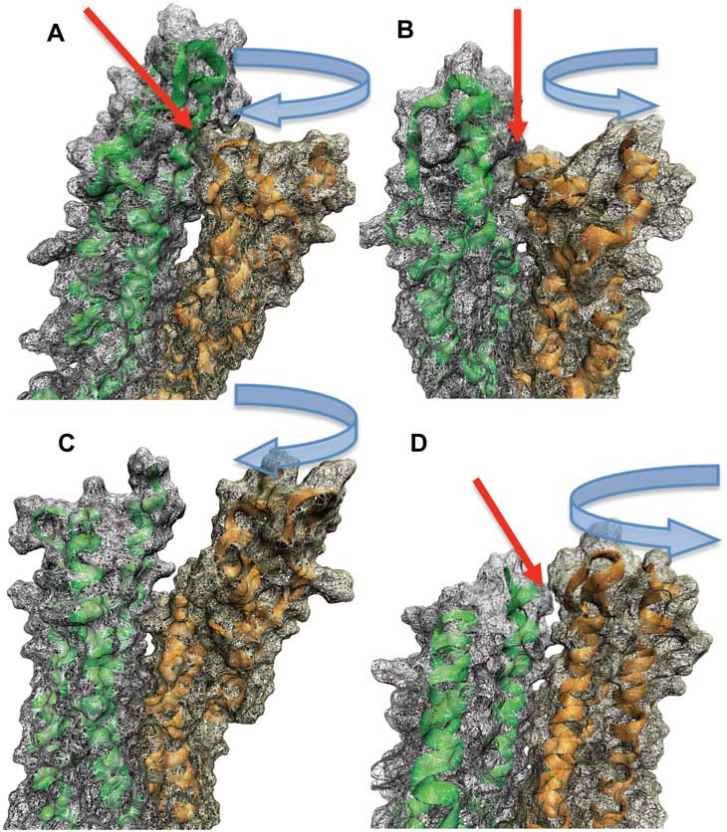
Steric interactions stabilize dimer in torsion simulations. A) VMD [52] rendered representation of the surface interactions (red arrow) at terminus B between the two monomers of the α-actinin rod domain after a 140-degree rotation in the clockwise direction. Steric interaction between the rotating monomer (orange) and the front surface of the other monomer (green) increase the torque required to continue rotation. B) Counterclockwise rotation at terminus B of the dimer conformation. Steric interactions occur (red arrow) after the rotation of one monomer (orange) around to the back surface of the other monomer (green). This steric interaction ends after about 170 degrees of rotation. C) Similar surface representation as in A and B but for Terminus A of the dimer conformation. One monomer (orange) is being rotated clockwise without any steric interactions. This is the only rotation of the dimer conformation that exhibits no steric interactions. D) Counterclockwise rotation at the Terminus A of the dimer. Steric interaction (red arrow) of the rotating monomer (orange) with the other monomer (green) occurs after only 60 degrees of rotation. Direction of rotation is indicated above each panel.

The trajectory of the α-actinin rod domain dimer under torsional simulation was largely a result of steric interactions between the rod domain monomers. The topology of R1 near terminus A is different from the topology of R4 near terminus B, and the steric interactions with each of their respective complementary repeats after rotation is different (Figure 8). Rotation of terminus A in the clockwise direction lacks steric interactions with the complementary monomer and thus requires less torque for rotation than does the counterclockwise rotation at terminus A (Figure 6 C). Counterclockwise rotation of terminus A results in steric interactions with the complementary monomer after only 60 degrees of rotation, and results in an increase in torque required for rotation after only 60 degrees of rotation. At terminus B both rotation in the clockwise and the counterclockwise direction result in steric interactions (Figure 8A and Video S4). The steric interactions occur when α-actinin is rotated beyond 150 degrees (Figure 6D). There is a greater resistance to clockwise rotation than counterclockwise rotation at terminus B because clockwise rotation involves both steric interactions and disruption of the aromatic packing.

**Figure 9:**
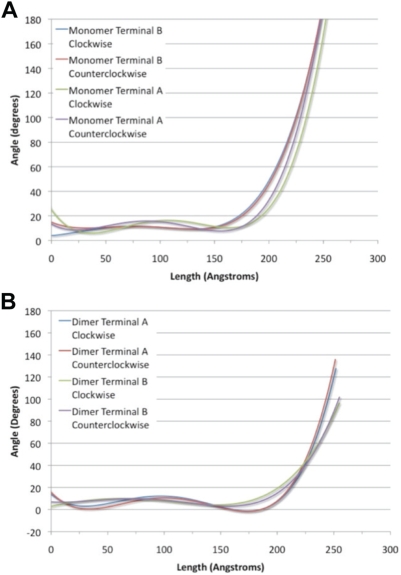
Distribution of torsion is localized to the termini of the molecule. A) 4^th^ order polynomial fit plot showing relationship between angle of rotation and distance from applied torque. Shown here is the torque applied to the dimer conformation at Terminus B (clockwise rotation in green, counterclockwise rotation in purple) and Terminus A (clockwise rotation in blue, counterclockwise rotation in red). Fixation constraints are placed at one terminus (the origin) and torsional constraints are placed at the other terminus (25 nm). Most of the rotation occurs locally near the terminus with the applied torque. B) Same plot as A for the rotation of the α-actinin dimer monomer. Clockwise rotation at Terminus B, shown in blue, clockwise rotation at Terminus A shown in green, counterclockwise rotation at Terminus B shown in red, and counterclockwise rotation at Terminus A shown in purple.

The results from torsional molecular dynamics simulations suggest that the dimerization of the α-actinin rod domain prevents rotation by introducing steric interactions that increase the torque needed to achieve rotation. Simulations of rotation of the monomer needed maximum 330 pN*nm of torque to rotate, while simulation of rotation of the dimer needed maximum 450 pN*nm (Figure S1). Aside from the steric interactions, the rotation of each monomer was resisted by aromatic packing interactions. The aromatic interaction had the additional effect of localizing most of the rotation to terminal regions (Figure 9). Normal mode analysis (Figure 2 and 3) also showed localization of the torsional movements to the terminal residues. Because of the strength of the aromatic interactions, it is likely more favorable for the α-actinin rod domain to further rotate terminal residues than to disrupt the aromatic packing and rotate other regions of the molecule. It is conceivable that the rotation may be localized to the terminal regions because of the speed of the rotation simulation, i.e. due to insufficient time for rotation to propagate to central regions as torque is applied. To test the effects of angular velocity on rotation we rotated the C-terminus of the two central repeats of the rod domain in the clockwise direction using two different rotational velocities: 0.5 degrees/ps and 0.05 degrees/ps (Figure S2). Rotation of the C-terminus of two central repeats in the clockwise direction at a slower rate showed a decrease in localization of conformational change to the terminus. Equilibration after rotation at terminus A did not, however, result in increased propagation of rotation (Figure S3). Both rotation at a slower rotational velocity and equilibration after rotation allow time for the torque to propagate to nearby residues. The aromatic packing interactions in repeat 4 however prevent propagation of rotation when torque is applied at terminus A, even after adequate equilibration after application of the torque (Figure S3). Comparison of the angular velocity used in the molecular dynamics simulations to the angular velocity calculated for torsional normal modes (∼0.005 degrees/ps) suggests rotation during molecular vibration is 100× slower than in our molecular dynamic simulation.

### Molecular Dynamics Simulation of Extension

Using constant-force molecular dynamics we explored the mechanical properties of α-actinin. Constant-force molecular dynamics is simulation of the conformational changes in a molecule resulting from application of a constant external force to specific residues. In the α-actinin simulations here, we apply a constant force to the C-terminus (terminus B) of one monomer and hold the other terminus fixed. Specific conformational changes are indicative of structural properties. Our simulations suggest that specific electrostatic interactions within each monomer and between the monomers contribute significantly to the stability of the rod domain dimer under extensional simulation.

**Figure 10:**
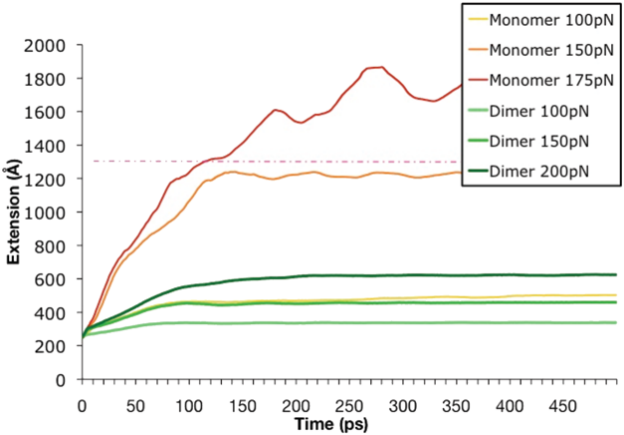
Dimerization increases the α-actinin rod domain rigidity especially in resilience to extensional forces. Terminus B of a monomer alone, and a monomer in dimer conformation were extended using external force. Results suggest that single monomers (yellow 100 pN, orange 151 pN, and red 175 pN) extend with less force than monomers in a dimer complex (green). Forces up to 200 pN were unable to fully extend the dimer complex but forces of 150 pN fully extended the single monomers. Full extension is indicated by the pink dotted line. Simulations were run for 500 ps and validated with shorter explicit simulations.

**Figure 11:**
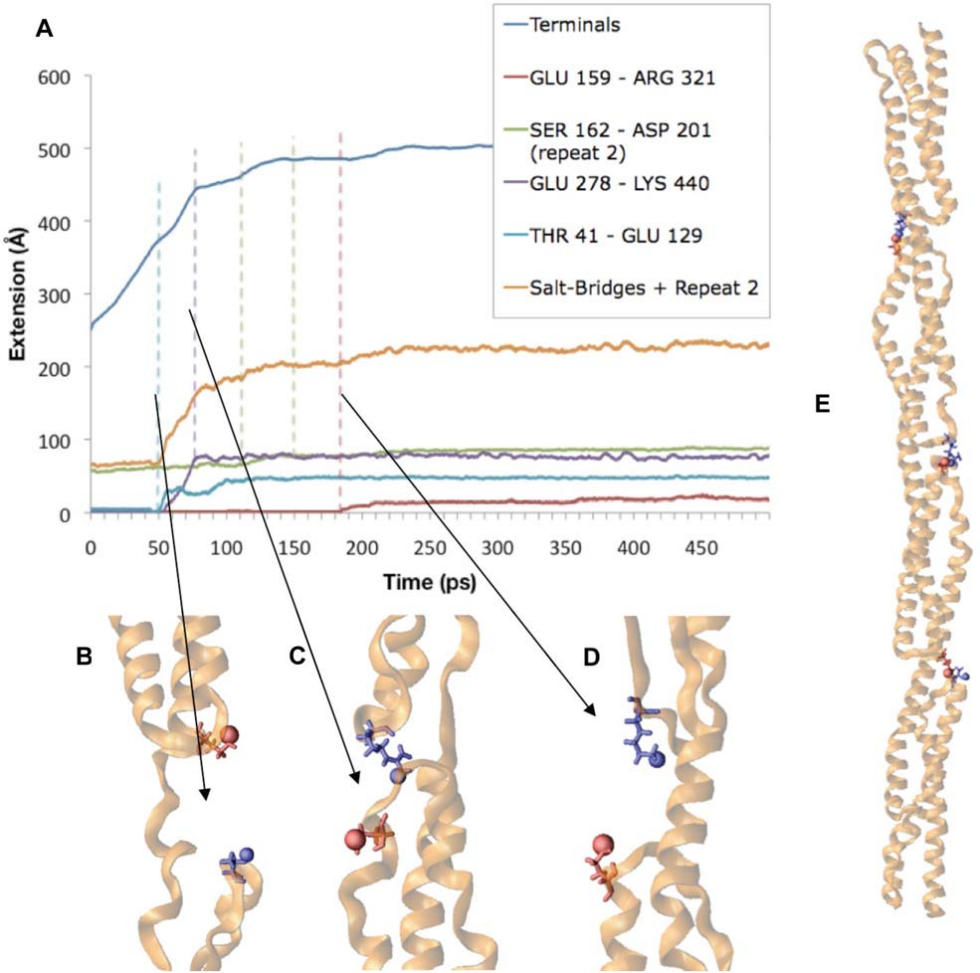
Extension of α-actinin monomer follows a trajectory dictated by the breaking of internal salt bridges. A) Extension with a 100 pN force. The trajectory of the extension of the termini of α-actinin (monomer) is shown in dark blue. The cumulative extension due to salt bridge interactions is shown in orange. Notice the breaking of the salt bridges corresponds directly with increases in the rate of the overall extension. Trajectories of three salt bridges are captured here: T41 and E129 (light blue), E278 and K440 (purple), and E159 and R321 (red). Each successive extension of the α-actinin molecule specifically corresponds to the breaking of one of these salt bridges. B) Image showing the breaking of T41 and E129. C) Image showing the breaking of E278 and K440. D) Image showing the breaking of E159 and R321. E) α-actinin monomer with the three salt bridges represented intact.

The α-actinin rod domain showed significant extensional rigidity. Forces were applied ranging from 100 pN to 200 pN to a monomer alone and to a monomer in dimer conformation, and only the monomer conformation with 150 pN or more of force was completely extended after simulation (Figure 10). Interactions within a single α-actinin rod domain stabilize the individual monomers under extension. Three key interactions dictate the extension trajectory of a single α-actinin monomer (Figure 11): T41-E129, E278-K440, and E159-R321. Studying the extension trajectory of an α-actinin rod domain monomer under a 100 pN force shows that every break in one of these three interactions corresponds to an increase in the extension rate of the monomer. Simulations with larger forces induce conformational changes at too rapid a rate to capture specific interactions. The simulation at 100 pN shows that before the T41-E129 interaction breaks, the extension of the monomer is due to helical regions near the terminus and linker helical regions. Once T41-E129 breaks (Figure 11B), the extension increases from helical unraveling in R1 and R2. The next big increase in extension occurs when the E278-K440 interaction breaks (Figure 11C). The extension continues and is once again extended with the extension and eventual breaking of the third key interaction E159-R321 (Figure 11D). The precise correlation of extension profile to salt bridge breakdown indicates the role these internal interactions play in stabilizing the individual monomers in the α-actinin rod.

**Figure 12:**
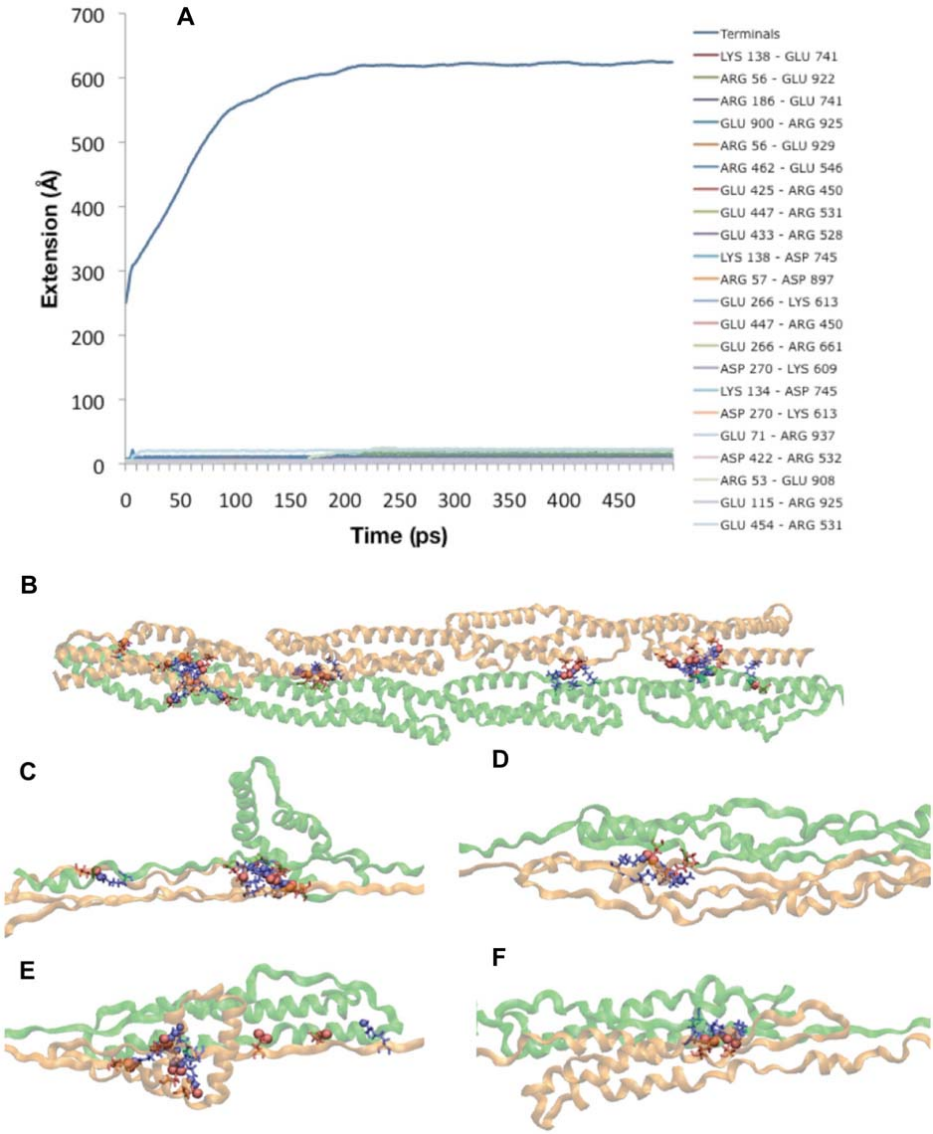
Extension of the α-actinin rod domain dimer. Extension of the α-actinin rod domain dimer was stabilized by 22 specific charged interactions between the two monomers. A) Plot showing the extension of the α-actinin rod domain dimer under 150 pN. Unlike the extension of a single α-actinin monomer, the trajectory of the extension of the dimer was largely dictated by helical extension not by disruption of charged interactions. B) α-actinin rod domain dimer with the 22 charged interactions stabilizing the dimer represented. C) Image showing the extension of R1 during simulation. Although the second monomer (green) is not forced, it too extends due to the salt bridges. D) Extension of R2 and associated R3 (green) under simulation. E) Extension of R3 and associated R2 (green) under simulation. F) Extension of R4 at terminus B where the extensional force is applied. Notice the charged interactions remain intact causing extension of the other monomer.

Dimerization increases the structural rigidity of α-actinin by introducing 22 specific charged interactions between the two monomers: E71-R937, R56-E929, R56-E922, E115-R925, R57-D897, E900-R925, R53-E908, K134-D745, K138-D743, K138-E741, R186-E741, E266-R661, E266-K613, D270-K613, D270-K609, E433-R528, E447-R531, E425-R450, E454-R531, E447-R450, D422-R532, and R462-E546. These interactions anchor one monomer under forced extension to the other monomer, increasing rigidity and reducing the length of extension. The extension profile of the α-actinin rod dimer (Figure 12 and Video S5) demonstrates that the salt bridges between the two monomers do not break as a result of the extensional force being applied. Furthermore, the extensional force is being applied to one monomer but in all four repeats (Figure 12 C–F) the extension is not limited to only the stressed monomer; the salt bridges cause extension in the associated monomers as well. The associated monomer extends as well because it is more energetically favorable to extend its helices than to break the salt bridges holding the two monomers together.

## Discussion

The actin filament cytoskeletal network is highly dynamic and stressed. Actin filaments are in continuous movement and rearrangements, and are also continuously exposed to external stresses. As an actin filament crosslinker, it is therefore functionally necessary for α-actinin to be both rigid and be able to withstand the external stress exposure, and to flexibly and dynamically scaffold the actin filaments. The structural mechanisms determining the necessary partial flexibility of α-actinin are as yet not fully understood. By applying methods of computational simulation and normal mode analysis we suggest several possible explanations for the molecular basis of the partial flexibility. Our results suggest that the α-actinin rod domain is flexible and dynamic near its termini while its central helical linkers are rigid, and its dimerized surface is highly stable. Our results also suggest that α-actinin has bending flexibility. Our molecular dynamics simulations suggest aromatic packing interactions play a role in resisting torsion, while electrostatic interactions play a role in resisting extension.

Our NMA study suggested that the most natural vibrational mode of the α-actinin rod domain, both for monomer and dimer conformations, is bending (Figure 2 and 3). This result was then further tested by our molecular dynamics simulations, which suggested bending forces as low as 24 pN could bend the α-actinin rod monomer. The NMA results suggested that extensional natural frequencies are at physiologically irrelevant higher frequencies and not in the low frequency normal modes (up to mode 18 at 21 Hz). Again, our molecular dynamics simulations suggested that extension of the α-actinin dimer requires extensional forces of over 200 pN. Physiologically, α-actinin needs to stabilize the actin filaments even if they are moving closer together, and the bending flexibility suggested by our study could contribute to α-actinin's ability to do so. These results are consistent with previous studies on DNA, myosin S2 region, and keratin, which also suggested that the coiled-coil structure allows for bending and torsional flexibility but has extensional rigidity [21]–[28].

Previous studies have shown through electron microscopy that α-actinin crosslinks actin filaments at numerous angles and lengths [35]. Their work suggests that although the actin filaments are continuously in lateral and horizontal movement, the α-actinin crosslinker is able to maintain its crosslink of the actin filaments. Our molecular dynamics simulations suggest that the α-actinin rod domain has bending flexibility, and that the rod domain is likely to bend while the crosslinker is under compressive stress. The exact direction of any physiological compressive stress is likely different from the bending forces used in our simulations, but the bending flexibility suggested by our simulations is likely the same.

The NMA results as well as the torsional molecular dynamics simulations suggested that the torsion of the molecule during its natural vibrations occurred mostly near its termini (Figure 9). Flexibility near the termini suggests that the flexible region of the α-actinin structure is its neck region, which connects the regions near the termini of the rod domain to the ABD and Cam domains (Figure 1). Flexibility in the neck region has been suggested several times, most recently by Sjoblom [43], and our results further suggest that the flexibility in that region is structurally facilitated by flexibility at the rod domain near its termini.

An interesting result from the bending simulations was the super coiling that occurred when bending a single α-actinin rod domain monomer (see Figure 4F). The monomer was bent in half and immediately arranged itself into a coiled-coil conformation. The bent monomer had several similarities to the dimer molecule; it was twisted 90 degrees, it had R1 interacting with R4, and R2 interacting with R3. The phenomenon suggests that the coiled-coil conformation is a result of the complementary surfaces on R1 and R4 and on R2 and R3.

The torsional studies pointed out the role the aromatic residues in each spectrin repeat play in stabilizing the α-actinin molecule. The aromatic packing play a critical role in resisting torsion and more importantly in restricting rotation to regions near the termini while the molecule is subjected torsion. The importance of the aromatic residues as suggested by our results is consistent with a previous study which suggested that aromatic residues in the spectrin repeats are conserved [8], and with previous studies on DNA which showed that aromatic packing plays a critical role in DNA structural characteristics [21]–[24].

Our extension results provide interesting insight into the mechanisms of dimer stabilization. The α-actinin rod domain was stabilized by both charged interaction within each monomer and by electrostatic interactions at the surface between the two monomers. Furthermore, the charged surface interactions relay extension on only one monomer into extension in the other monomer. Several previous studies are consistent with this phenomenon and our results strengthen these past studies. Law et al. [44] studied the interaction of α-spectrin and ß-spectrin in dimerization while external forces were applied. They suggested that the regions with stronger intermonomer interactions had unfolding at both monomers, whereas regions with weaker intermonomer interactions had only unfolding in one monomer. Ortiz et al. [45] studied the effects of hydration on linker stability. They found that once the molecular regions blocking off the linker from hydration were broken, the linker lost its conformation. We find in our study that the interaction preventing hydration of the linker region in α-actinin is the electrostatic interactions between charged residues in front of the linker regions (see Figure 11). Other studies [13],[46] have suggested that the mechanisms for stabilization of the α-actinin dimer are electrostatic interactions on the surface and acidic residues on the surface. Our results suggest that the charged interactions on the surface between the monomers play a vital role in providing rigidity.

The normal mode analysis results were determined using the WEBnm@ [44] method which shows global conformational movements of the rod domain. In contrast, the molecular dynamics simulations of rotation, bending, and extension, all showed local conformational changes determined by interactions at the atomic level. The results from both sets of investigations are similar and therefore suggest that the global conformational changes seen in the normal modes are correlated to local atomic interactions seen in the molecular dynamics simulations.

Results from our study suggest the α-actinin rod domain is stabilized by electrostatic interactions between the monomers, aromatic interactions within each monomer, and steric interactions between the monomers. The results also suggest that the rod domain has bending flexibility, torsional flexibility near its termini, and extensional rigidity. These mechanical properties could play a role in facilitating α-actinin's role as an actin filament crosslinker. Experimental investigations can further test some of the results presented here. Our results suggest that mutation of aromatic residues would reduce resistance to torsion, and mutation of electrostatic residues would reduce extensional rigidity. Mutagenesis of such residues, therefore, could reduce the mechanical stability of α-actinin and decrease its effectiveness as an actin filament crosslinker.

## Materials and Methods

### Normal Mode Analysis (NMA)

Natural frequencies of both the monomer of the α-actinin rod and its dimer form were determined using WEBnm@ [47]. WEBnm@ is an online normal mode analysis tool developed using the computational methods of Hinsen [48]. Normal modes are calculated by determining the eigenvectors of the matrix of second derivatives of energy with respect to displacement of the Cα atoms of each residue. Because NMA represents movements resulting from overall structure, the use of Cα force fields are sufficient for NMA calculations [49]. WEBnm@ uses MMTK [50] toolkits internally and provides a web graphical user interface for implementing MMTK scripts. MMTK is an open source library of molecular modeling scripts developed in the PYTHON programming language. The 6 lowest frequency modes of α-actinin represent the translational and rotational normal modes and are ignored since these modes involve whole protein movement and demonstrate no conformational dynamics [51]. The next 6 lowest frequency normal modes (modes 7–12) exhibit the lowest energy natural vibrational frequencies and conformational movement. The natural frequencies are a property of the molecular structure and can be used to differentiate rigid molecular regions from flexible molecular regions. The natural frequencies also reveal movements (such as bending, torsion, or extension) that are natural to the molecular structure of α-actinin.

The crystal structure for the α-actinin rod domain (PDB ID = 1HCI) [15] was retrieved from the Protein Data Bank (PDB) and used for NMA by WEBnm@ after 1000 steps of the Adopted Basis Newton-Raphson (ABNR) method for minimization in CHARMm [51]. The NMA simulation with WEBnm@ uses only Cα force fields and calculates normal modes without a solvent environment. Resulting vibrational frequencies were visualized using the molecular visualization software VMD [52]. Vector fields of the vibrational movements were produced by WEBnm@ and used in VMD along with a new ribbon representation to illustrate the modal movements. WEBnm@ also calculated the individual residue vibrational movements using the RMSD methods of Schulz [53]. NMA analysis was carried out separately for both the monomer (spectrin repeats 1–4) and dimer (8 spectrin repeats).

### Simulation of Force-Induced Bending

Molecular dynamics simulations were carried out using the commercially available software CHARMm [54]. α-actinin structure (PDB ID = 1HCI) was retrieved from PDB and minimized using the Adopted Basis Newton-Raphson (ABNR) method for 1000 steps. For equilibration and simulation the implicit water solvation model ACE [55] was used. After minimization, each simulation was heated to 310 K and equilibrated using the VERLET loop function in CHARMm. Throughout the equilibration and bending simulation the temperature was controlled using the Hoover temperature control method [56]. Charmm22 force field definitions [57] were used along with the SHAKE method [58] for applying harmonic constraints on the bond lengths to hydrogen atoms. All molecular dynamics simulations were carried out with 1 fs timesteps and simulations were run for 500,000 timesteps (500 ps). Results were visualized using VMD [52].

Bending simulations were carried out on both dimer conformations (8 spectrin repeats) of α-actinin and monomer conformations (4 spectrin repeats). For the simulations on the monomer conformations, constraints were placed on the α-carbon of I320, S162, and L240, the three central residues suggested to be hinges in the NMA studies. Bending forces were applied to S1 and N85 at terminus A and to G398 and D475 at terminus B. Vector direction of the bending forces was taken directly from the bending normal modes. Total bending force applied to the α-actinin monomer ranged from 8 pN to 200 pN.

Bending simulations on the dimer conformation were carried out similarly. Constraints were placed on the α-carbon of S161, L240, I320, S637, L715, and S798. Bending forces were applied to S1, N85, H873, and D950 at terminus A and G398, D475, S476, and N560 on terminus B. Total bending force applied to the α-actinin dimer ranged from 24 pN to 200 pN.

### Molecular Dynamics Simulation of Torsion

Α-actinin crystal structure with PDB ID = 1HCI [15] was solvated in a water box with a 10 Å radius of solvation. For the dimer conformation with 950 residues about 100,000 water molecules were added. For the monomer conformation with 475 residues about 65,000 water molecules were added. Molecular dynamics were carried out with NAMD [59]. Each structure was minimized for 1000 steps and equilibrated for 400,000 steps (400 ps). Equilibration and simulations were run with 1 fs timesteps. Results were visualized using VMD [52].

Torsion was implemented using the rotating constraints function of NAMD using the CHARMM22 force field definition [57]. The target atom to be rotated was attached to a reference atom with a spring of known stiffness K. The reference atom was rotated at a known angular velocity about the axis of rotation and the resulting conformational changes in α-actinin were determined by the molecular dynamics. If ***ν*** is the unit vector of the axis of rotation, M the rotation matrix, **P** the coordinates of the pivot point, **R** the coordinates of the reference atom, and **R_0_** the original coordinates of the reference atom, then the location of the reference atom can be determined by **R** = M(**R_0_**−**P** )+**P**. If **X** is the location of the target atom, then the normal is defined as **N** = (**P**+((**X**−**P**) ***^.^ν***)***ν***)−**X**. The force applied to the target atom by the spring attaching it to the rotating reference atom can be calculated as **F** = 2 K(**R**−**X**). The torque is then calculated as torque = **F**×**N**.

The torsion simulations were carried out with rotating constraints applied to terminus B or terminus A, and rotated the target atoms in either the clockwise or the counterclockwise direction. For simulations on both the α-actinin monomer and dimer rotated at terminus B, fixation constraints were placed on residues 1, 2, 3, 4, 5, 84, 85, and 86 at terminus A, and rotating constraints were placed on residues 396, 397, 398, 399, 400, 401, 469, 470, 471, 472, 473, 474, and 475 at terminus B (Figure 5). For simulations on the α-actinin monomer and dimer rotated at terminus A, fixation constraints were placed on residues 396, 397, 398, 399, 400, 401, 469, 470, 471, 472, 473, 474, and 475 at terminus B, and rotating constraints were placed on residues 1, 2, 3, 4, 5, 84, 85, and 86 at terminus A (Figure 5). Residue 1 and 475 are also part of the axis of rotation, and application of rotation constraints to these residues has the purpose of inducing rotation to these residues once they move off the axis of rotation due to conformational changes induced by rotation of nearby residues. For simulations with rotation targeted at terminus B, the pivot for the axis of rotation was at the α-carbon of residue 1 and the axis was defined in the direction from residue 1 to residue 475. For simulations with rotation targeted at terminus A, the pivot for the axis of rotation was placed at the α-carbon of residue 475 and the axis of rotation defined in the direction from residue 475 to residue 1. The specific axis of rotation used in the molecular dynamics simulations was chosen to be consistent with rotation as seen in normal mode analysis. Normal mode analysis of the monomer (Figure 2) suggested that the residues near the termini rotate around the axis running through the center of the molecule. The axis from residue 1 to residue 475 is set up to also run through the center of the molecule. For all simulations a spring stiffness of K = 10 Kcal/molÅ^
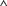
^2 was used. The reference atoms were rotated at a constant angular velocity of 0.005 degrees per timestep (0.5 degrees/ps) in the positive direction for counterclockwise simulations, and in the negative direction for clockwise simulations. Simulations were run for 400 ps to achieve near 180-degree rotation. To illustrate the effects of rotation speed, the C-terminus of the two central repeats of α-actinin were rotated clockwise at two different rotational speeds: 0.005 degrees per timestep (0.5 degrees/ps) and 0.0005 degrees per timestep (0.05 degrees/ps) (Figure S2). Quantitative results of applied torque and angle of rotation were calculated using MATLAB (2007a, The Mathworks, Natick, MA). The average torque applied on each residue during simulation is reported below. Torque values have been interpolated using a fourth-order polynomial fit to reveal trends from the oscillations in applied torque values.

### Constant Force Molecular Dynamics Simulation of Extension

Extension studies were carried out first in implicit solvent using CHARMm [54] and later verified using explicit simulations in NAMD [59]. α-Actinin atomic coordinates were taken from the crystal structure PDB ID = 1HCI [15]. For implicit solvent simulation of the α-actinin molecule the ACE [55] implicit solvent method was used. For the explicit solvent simulations a water box with a solvation radius of 15 angstroms was elongated to 48.8 nm×17.2 nm×8.5 nm to ensure solvation of an extended α-actinin. The resulting water box had over 230,000 water molecules. The cut-off length of non-bonded interactions was set to 12 Å. Explicit solvent simulation was run for 100 ps with 100 pN of force, and a timestep of 1 fs. Forced extension simulations were carried out on a monomer conformation with only the four spectrin repeats, a dimer conformation with 8 total spectrin repeats, and single spectrin repeat (R1) created based on the repeat definitions given by Gilmore et al. [60]. The results were visualized using VMD [52].

For accurate and efficient simulation the hydrogen atom bond length was constrained using SHAKE [58]. The SHAKE method fixes bond lengths between large atoms and hydrogen atoms preventing unnecessary calculation of irrelevant interactions. One fs timesteps were used in both implicit and explicit simulations, and simulations were run at 310 K. The α-actinin molecule was minimized using ABNR for 1000 steps and equilibrated using the VERLET loop function of CHARMm. Charmm 22 force field [57] definitions were used. Constant forces ranging from 100–200 pN were applied to the terminus B α-carbon at residue 475, and fixation constraints were applied to the α-carbon of residue 1 at terminus A in both the monomer and dimer simulations. Forces were applied in the vector direction from terminus A to terminus B. The implicit solvation simulations were run for 500 ps, and the explicit solvation simulations were run for 100 ps for verification.

## Supporting Information

Figure S1 Effects of dimerization on torque required for rotation of α-actinin. The α-actinin rod domain monomer and dimer were exposed to external torsional stress in both the clockwise and the counterclockwise directions. (A) Torsion applied at terminus A required torque of up to 350 pN*nm to rotate the monomer and torque of up to 400 pN*nm to rotate the dimer. Rotation beyond 140 degrees correlated with an increase in torque required for rotation of the dimer and the monomer in the clockwise direction, but not the monomer in the counterclockwise direction. Steric interactions between two monomers in the dimer conformation can account for the increase in torque in the dimer conformation. (B) Rotation at terminus B required torque of up to 350 pN*nm to rotate the monomer and torque of up to 450 pN*nm to rotate the dimer. The significant increase in rotation of the dimer conformation at terminus B can also be explained by steric interactions. In both plots, rotation of the dimer in the clockwise direction is shown in blue, dimer in the counterclockwise direction is shown in red, monomer in the clockwise direction is shown in green, monomer in the counterclockwise direction is shown in purple.
(2.13 MB TIF)

Figure S2 Effects of rotational velocity on the α-actinin rod domain central repeats. The two central repeats of the α-actinin rod domain monomer were rotated at the C-terminus in the clockwise direction at two different rotational velocities. The C-terminal residues of the rod domain were rotated at 0.5 degrees/ps (blue) and at 0.05 degrees/ps (red). Results show that rotation at the slower rotational velocity decreases the localization of rotation to the C-terminus. Residues further from the C-terminus undergo more rotation at the slower rotational velocity than at the faster rotational velocity. At both rotational velocities the residues near the N-terminus of the two central repeats under simulation are do not undergo appreciable rotation.
(0.78 MB TIF)

Figure S3 Equilibration of α-Actinin after rotation and propagation of torque. Rotation of the α-actinin rod domain dimer at terminus B was extended for a 100 ps equilibration. Static harmonic constraints were used in the place of the rotating harmonic constraints. The rotated conformation was then equilibrated for 100 ps. The rotation of each residue is plotted against the distance of the residue form terminus A at the end of the rotation simulation and after the 100 ps equilibration. Both before equilibration (blue curve) and after equilibration (green curve) show localization of rotation to residues near terminus B. Aromatic packing interactions prevent propagation of torque to further residues.
(0.26 MB TIF)

Table S1 Comparison of the vibrational normal modes in the α-actinin rod domain. The lowest frequency vibrational normal modes of the α-actinin rod domain monomer and dimer were calculated using WEBnm@ [44]. Vibrational movement at the lowest frequencies correlates to conformational changes in the rod domain that are likely to occur. The lowest frequency vibrational movement of both the rod domain monomer and the rod domain dimer can be characterized as one-hinge bending (see Figure 2 and Figure 3). Other vibrational movements in the low frequency normal modes of both the dimer and monomer conformations include: torsion at the termini, and three-hinge bending movements. Higher frequency normal modes (mode 18 and beyond in the monomer, and mode 16 and beyond in the dimer) consist of high-energy conformational changes that are not relevant to the structural analysis of α-actinin.
(5.82 MB TIF)

Video S1 Torsional vibrational movement in the α-actinin rod domain monomer. Normal mode analysis of the α-actinin rod domain monomer showed torsional movement at some of the modal frequencies. Torsional movement occurs at the residues near the termini. Shown here is a video of the torsional movement at the termini during vibration in mode 11. Residues in the central repeats undergo little conformational change while residues near the termini experience the rotational conformation change.
(0.22 MB MOV)

Video S2 Torsional vibrational movement in the α-actinin rod domain dimer. Normal mode analysis of the α-actinin rod domain dimer conformation showed several torsional modes. The torsional modes in the dimer conformation occur near the terminal residues and little rotation occurs at the residues in the central repeats. This video shows that rotational movement occurs at the residues near the termini and not at the central repeat residues.
(0.11 MB MOV)

Video S3 Rotation of the α-actinin monomer with molecular dynamics. Residues at both terminus A and terminus B of the α-actinin rod domain monomer were rotated in the clockwise and the counterclockwise directions. Shown here is rotation of the rod domain monomer terminus B in the clockwise direction. Aromatic residues are shown in orange and other hydrophobic residues are shown in yellow. During rotation a specific aromatic packing interaction between aromatic residues W381 and Y417 is maintained until rotation passed about 140 degrees. Once terminus B is rotated beyond 140 degrees the aromatic packing interaction is broken. Breaking of the aromatic packing interaction corresponds to an increase in torque required for rotation beyond 140 degrees (see Figure 6A).
(5.67 MB MPG)

Video S4 Rotation of the α-actinin rod domain dimer with molecular dynamics. One of the monomers in the α-actinin rod domain dimer was rotated using molecular dynamics. Rotation at both termini showed that the direction of rotation affects the torque required to achieve rotation (Figure 6B). Differences in torque required to achieve rotation arise from steric interactions between the monomers resisting rotation of one of the monomers. Shown here is rotation at terminus B in the clockwise direction. Rotation of one monomer (pink) involves both breaking of aromatic interactions (orange) and steric interaction with the other monomer (green). Rotation beyond 150 degrees introduces the steric interactions seen here and correlates to a peak in torque required to achieve rotation (Figure 6B).
(7.95 MB MPG)

Video S5 Extension of the α-actinin rod domain dimer with molecular dynamics. External force of 150 pN was applied to terminus B of a monomer of the α-actinin rod domain dimer. Forced extension of a single monomer caused extension of its complementary monomer as well due to several salt bridges between the two monomers. Shown here is the extension of one monomer (pink) and the resulting extension of the other monomer (blue). Throughout the simulation, the electrostatic interactions between the monomers are resilient and remain intact. Because it is more energetically favorable, the complementary monomer undergoes extension and conformational change but the electrostatic interactions remain intact.
(4.65 MB MOV)
