# Supplementary figures and images for: Molecular Mechanics of the α-Actinin Rod Domain: Bending, Torsional, and Extensional Behavior
Source: PLoS Comput Biol. 2009 May 15;5(5):e1000389. doi: 10.1371/journal.pcbi.1000389 (PMC2676514; doi:10.1371/journal.pcbi.1000389)

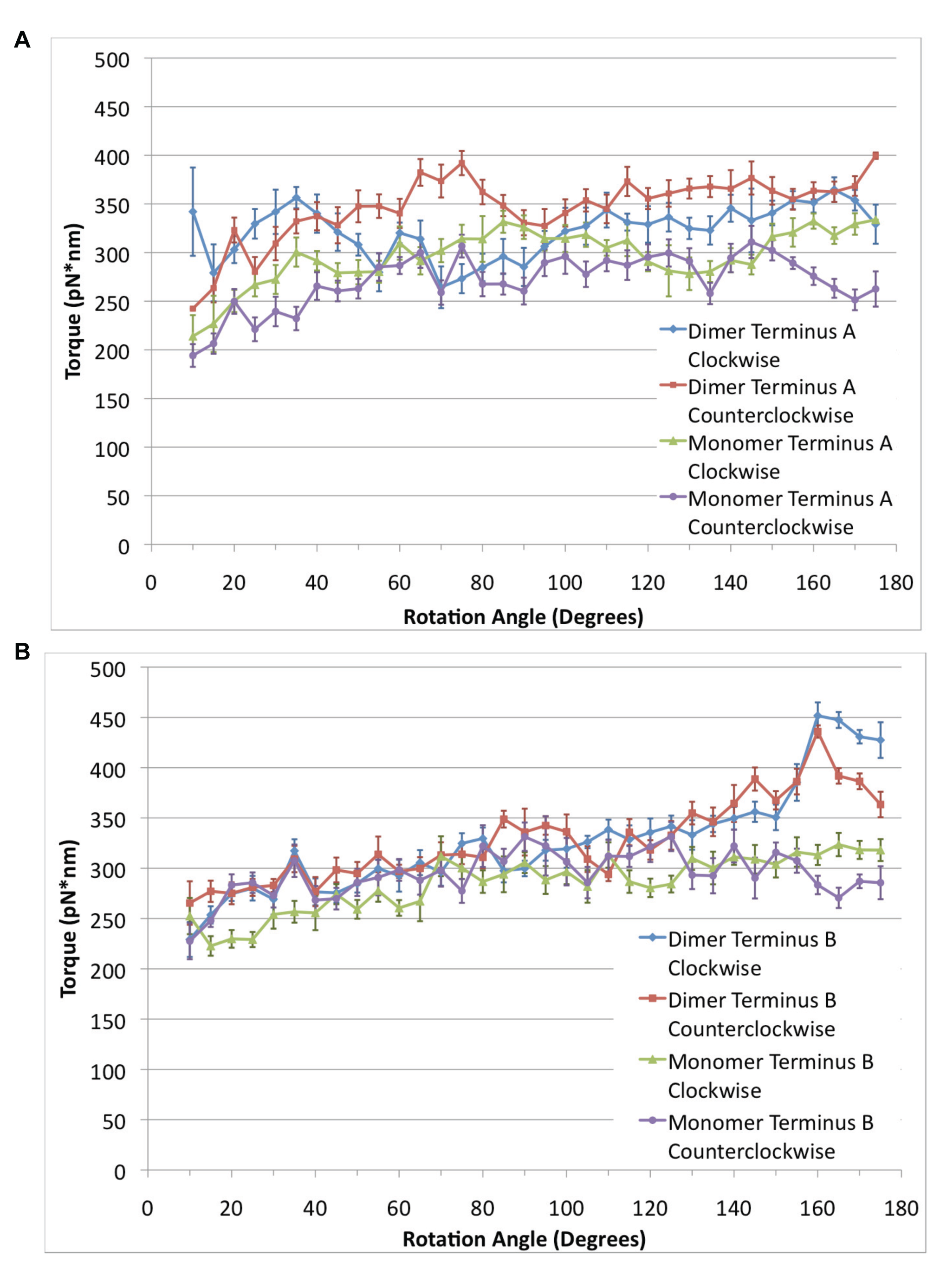

Supplement: Figure S1 — Effects of dimerization on torque required for rotation of α-actinin. The α-actinin rod domain monomer and dimer were exposed to external torsional stress in both the clockwise and the counterclockwise directions. (A) Torsion applied at terminus A required torque of up to 350 pN*nm to rotate the monomer and torque of up to 400 pN*nm to rotate the dimer. Rotation beyond 140 degrees correlated with an increase in torque required for rotation of the dimer and the monomer in the clockwise direction, but not the monomer in the counterclockwise direction. Steric interactions between two monomers in the dimer conformation can account for the increase in torque in the dimer conformation. (B) Rotation at terminus B required torque of up to 350 pN*nm to rotate the monomer and torque of up to 450 pN*nm to rotate the dimer. The significant increase in rotation of the dimer conformation at terminus B can also be explained by steric interactions. In both plots, rotation of the dimer in the clockwise direction is shown in blue, dimer in the counterclockwise direction is shown in red, monomer in the clockwise direction is shown in green, monomer in the counterclockwise direction is shown in purple. (2.13 MB TIF) [file pcbi.1000389.s001.tif]

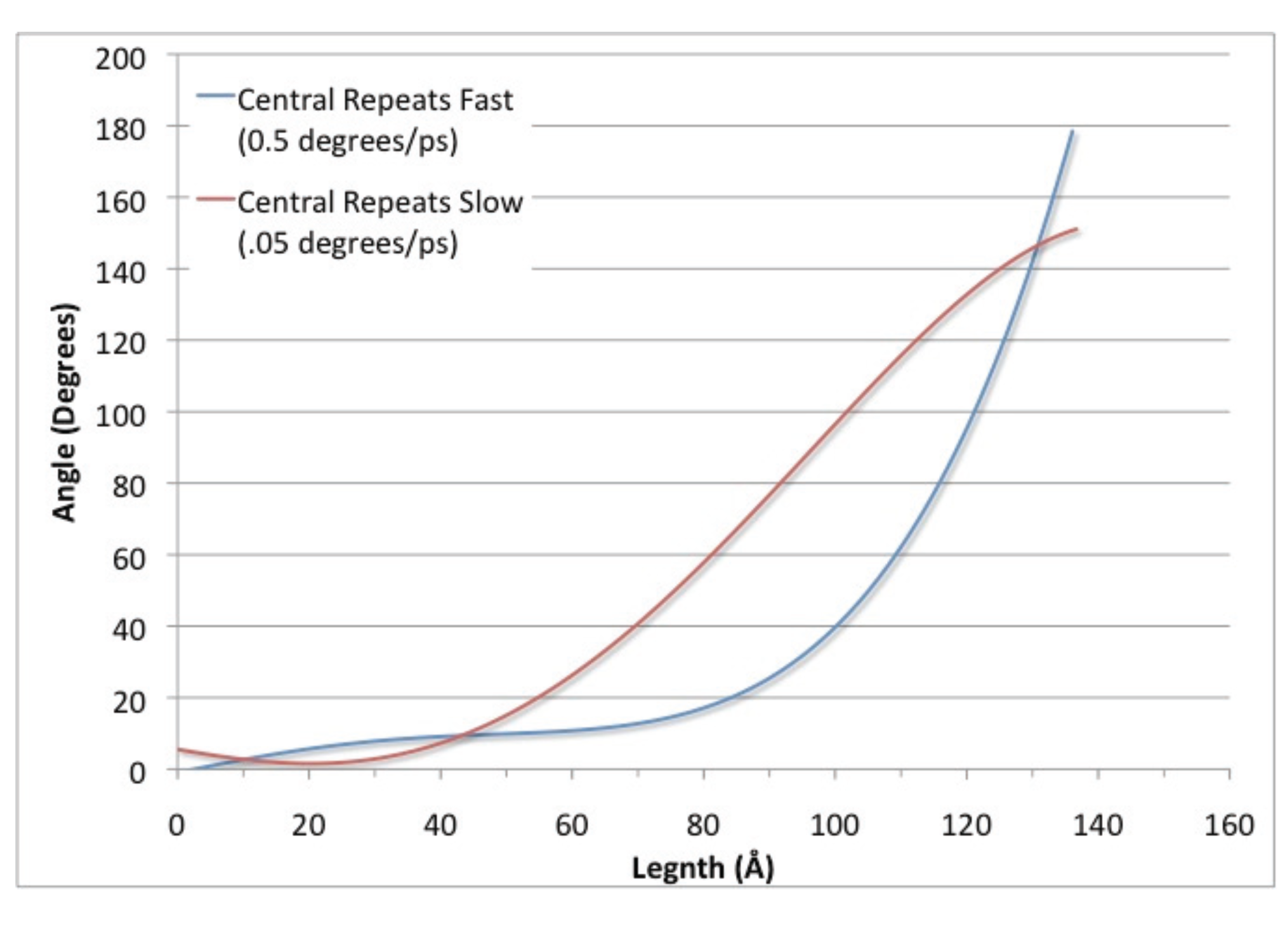

Supplement: Figure S2 — Effects of rotational velocity on the α-actinin rod domain central repeats. The two central repeats of the α-actinin rod domain monomer were rotated at the C-terminus in the clockwise direction at two different rotational velocities. The C-terminal residues of the rod domain were rotated at 0.5 degrees/ps (blue) and at 0.05 degrees/ps (red). Results show that rotation at the slower rotational velocity decreases the localization of rotation to the C-terminus. Residues further from the C-terminus undergo more rotation at the slower rotational velocity than at the faster rotational velocity. At both rotational velocities the residues near the N-terminus of the two central repeats under simulation are do not undergo appreciable rotation. (0.78 MB TIF) [file pcbi.1000389.s002.tif]

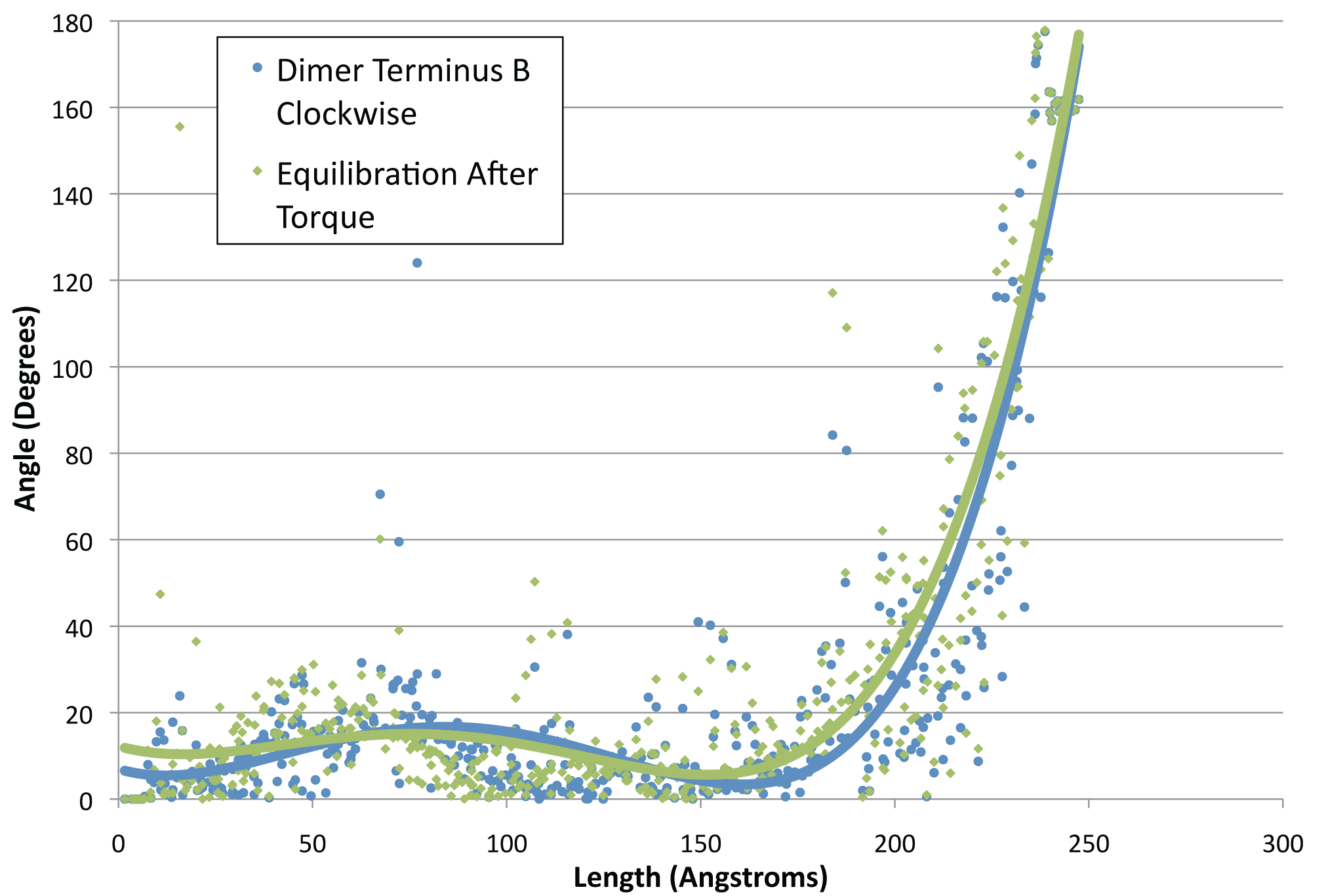

Supplement: Figure S3 — Equilibration of α-Actinin after rotation and propagation of torque. Rotation of the α-actinin rod domain dimer at terminus B was extended for a 100 ps equilibration. Static harmonic constraints were used in the place of the rotating harmonic constraints. The rotated conformation was then equilibrated for 100 ps. The rotation of each residue is plotted against the distance of the residue form terminus A at the end of the rotation simulation and after the 100 ps equilibration. Both before equilibration (blue curve) and after equilibration (green curve) show localization of rotation to residues near terminus B. Aromatic packing interactions prevent propagation of torque to further residues. (0.26 MB TIF) [file pcbi.1000389.s003.tif]

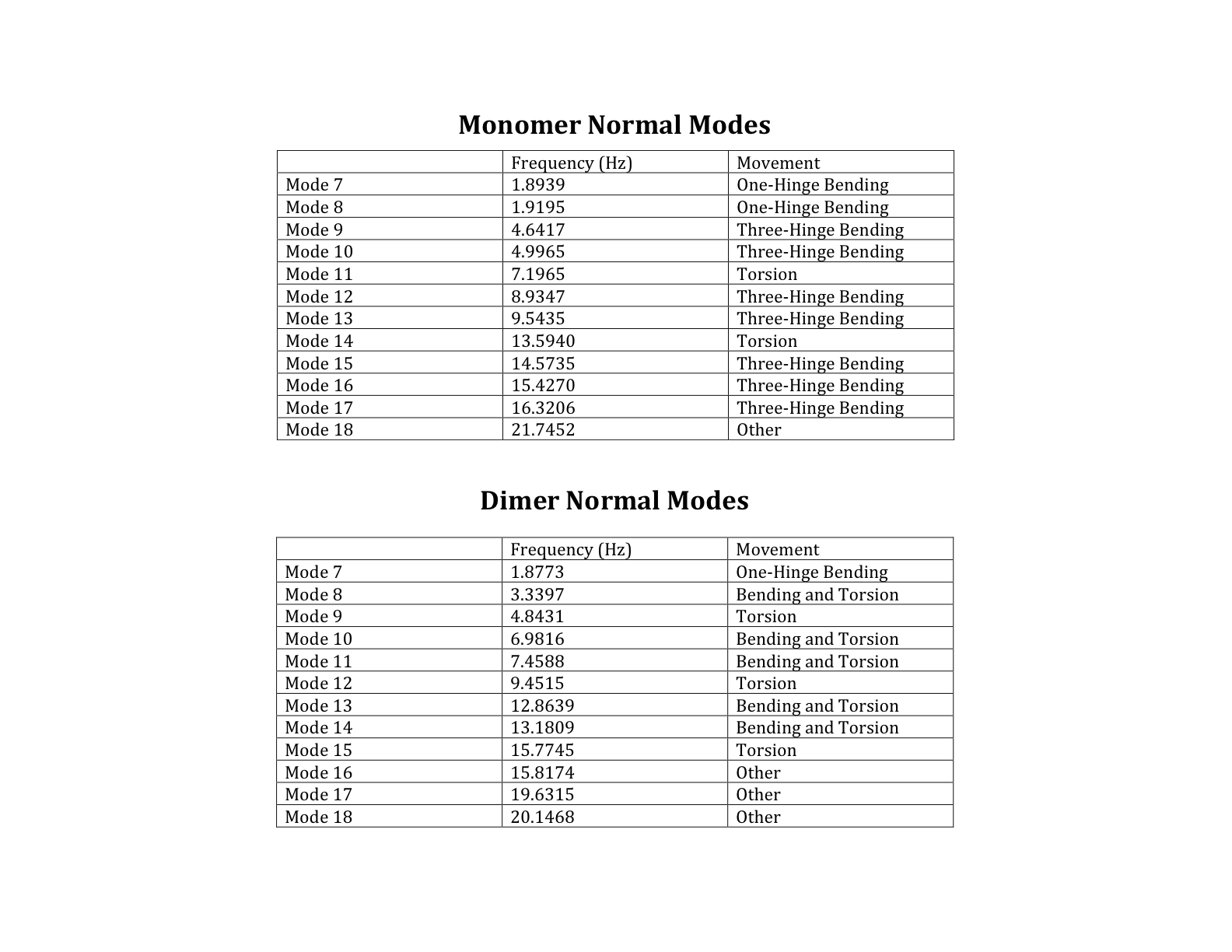

Supplement: Table S1 — Comparison of the vibrational normal modes in the α-actinin rod domain. The lowest frequency vibrational normal modes of the α-actinin rod domain monomer and dimer were calculated using WEBnm@ [44]. Vibrational movement at the lowest frequencies correlates to conformational changes in the rod domain that are likely to occur. The lowest frequency vibrational movement of both the rod domain monomer and the rod domain dimer can be characterized as one-hinge bending (see Figure 2 and Figure 3). Other vibrational movements in the low frequency normal modes of both the dimer and monomer conformations include: torsion at the termini, and three-hinge bending movements. Higher frequency normal modes (mode 18 and beyond in the monomer, and mode 16 and beyond in the dimer) consist of high-energy conformational changes that are not relevant to the structural analysis of α-actinin. (5.82 MB TIF) [file pcbi.1000389.s004.tif]
